# The Effect of Variations in Temperature and Contact Time of Zingerone, [6]-Gingerol and Shogaol as Disinfectants on *Staphylococcus aureus*, *Escherichia coli*, *Pseudomonas aeruginosa* and *Candida albicans*

**DOI:** 10.3390/microorganisms14030539

**Published:** 2026-02-26

**Authors:** Fathy A. A. Hasan, Abdullahi Umar Ibrahim, Kaya Suer, Suleyman Asir

**Affiliations:** 1 Department of Biomedical Engineering, Near East University, Mersin 10, Nicosia 99138, Turkey; ftheboosa@gmail.com (F.A.A.H.); suleyman.asir@neu.edu.tr (S.A.); 2 Department of Medical and Clinical Microbiology, Near East University, Mersin 10, Nicosia 99138, Turkey; kaya.suer@neu.edu.tr

**Keywords:** Gingerol, Shogaol, disinfectants, Gram-positive bacteria, Gram-negative bacteria, fungi

## Abstract

Rising microbial resistance to synthetic disinfectants has intensified the search for chemically synthesized natural alternatives, such as ginger-derived bioactive compounds. Several bioactive compounds, including Zingerone, Gingerols, and Shogaols, have been shown to possess antimicrobial activities. However, the antimicrobial efficacy of these compounds as disinfectants at varying temperatures and contact times is poorly understood. Therefore, understanding the temperature- and time-dependent effects of contact is crucial for optimizing the potential application of these compounds in various antimicrobial strategies. In this study, the antimicrobial activities of three chemicals, 10% [6]-Gingerol, Zingerone, and Shogaols, were evaluated against *Staphylococcus aureus*, *Escherichia coli*, *Pseudomonas aeruginosa*, and *Candida albicans*. Furthermore, the variations in temperatures (5 °C, 25 °C, and 37 °C) and contact time (1, 5, and 60 min) were assessed based on CFU counts, log_10_ reductions, percent kill, and decimal reduction time. The findings of this study indicated that 10% Zingerone completely inactivated all tested organisms in 60 min at all temperatures. Although 10% [6]-Gingerol and Shogaol exhibited temperature- and time-dependent effects, they failed to completely inactivate the bacteria and fungi after 60 min. Furthermore, both temperature and contact time were shown to influence the efficacy of the tested disinfectants, providing a significant time- and temperature-dependent reduction in viable cells across all tested organisms.

## 1. Introduction

The COVID-19 pandemic significantly increased the use of biocides, highlighting their role in infection control but also exacerbating concerns over antimicrobial resistance (AMR) and environmental toxicity associated with synthetic disinfectants, such as quaternary ammonium compounds (QACs) and alcohols [1,2,3,4,5,6]. While effective, these agents face limitations, including the potential to drive AMR, reduced efficacy in the presence of organic matter, and safety issues [7,8,9,10,11,12,13]. Plant-based alternatives offer eco-friendly profiles but often suffer from variable composition, standardization challenges, and lower potency [14,15,16,17].

Ginger (*Zingiber officinale Roscoe*) is a prominent source of bioactive compounds with demonstrated antimicrobial properties. Key constituents include Zingerone, Gingerols (e.g., [6]-Gingerol), and Shogaols, which are formed from Gingerols via dehydration [18,19]. These compounds exhibit a range of pharmacological activities, including antimicrobial effects against various bacteria and fungi [20,21,22,23,24,25,26,27]. For instance, ginger extracts and specific compounds like [6]-Gingerol have shown activity against pathogens such as *Staphylococcus aureus*, *Escherichia coli*, *Pseudomonas aeruginosa*, and *Candida albicans* [23,24,27].

Shogaols, Zingerone, and Gingerols are some of the disinfectants reported in the literature. Zingerone, chemically known as vanillylacetone, is a naturally occurring chemical compound derived from ginger (*Zingiber officinale*). The plant-derived compound is associated with several pharmacological properties, including antimicrobial, antioxidant, anti-inflammatory, antidiabetic, anxiolytic, and appetite stimulant effects [28]. Zingerone antimicrobial activity involves its ability to inhibit the growth of certain microbes. Thus, the compound has great potential as a remedy for various infectious diseases. However, further studies are required to fully understand its mechanism of action and pharmacological potency [28,29].

Like Zingerone, Gingerol is another plant-based compound derived from *Zingiber officinale*. Gingerols contain several phenolic compounds, including [6]-Gingerol, [8]-Gingerol, and [10]-Gingerol. The compound is associated with the spicy flavor of *Zingiber officinale* and several pharmacological activities, including antimicrobial, antioxidant, anti-cancer, and anti-inflammatory effects [20,30,31].

Shogaols are another class of chemical compounds derived from *Zingiber officinale*, produced by dehydrating Gingerols during processing or storage. Shogaols differ from Gingerols based on the replacement of hydroxyl groups (Gingerols) with ketone groups. There are several forms of Shogaols, including 4-, 6- (regarded as the most potent), 8-, 10-, and 12- Shogaols. These compounds are associated with part of ginger’s pungent flavor and numerous medicinal and biological properties, including antimicrobial, antioxidant, and anti-inflammatory effects [32,33,34].

Chemically synthesized versions of natural compounds, such as those derived from ginger, offer potential advantages including consistent quality, scalable supply, and the ability to study structure/activity relationships. However, empirical data are lacking on their performance under varied environmental conditions. Moreover, the practical application of disinfectants is critically influenced by operational factors such as contact time and temperature. Deviations from recommended contact times or use at non-standard temperatures can drastically reduce efficacy and promote resistance [35,36,37,38]. Therefore, there is a pressing need for effective disinfectants that maintain activity across a range of realistic use conditions. To address this gap, this study aimed to carry out the following:

- Investigate the efficacy of three synthetic ginger-derived compounds (Zingerone, [6]-Gingerol, and Shogaol) as alternative disinfectants.
- Evaluate their activity against representative Gram-positive (*S. aureus*) and Gram-negative (*E. coli*, *P. aeruginosa*) bacteria, and the fungus *C. albicans*.
- Systematically assess the impact of temperature (5 °C, 25 °C, 37 °C) and contact time (1, 5, 60 min) on antimicrobial efficacy.
- Generate empirical data to inform potential real-world applications in healthcare, the food industry, or environmental settings.

## 2. Materials and Methods

### 2.1. Materials

Chemical reagents: Zingerone (100 mg: Cat. No.: HY-14621.CAS No.: 122-48-5. Molecular Formula: C11H14O3. Molecular Weight: 194.23), [6]-Gingerol (100 mg: Cat. No.: HY-14615. CAS No.: 23513-14-6. Molecular Formula: C17H26O4. Molecular Weight: 294.39), and Shogaol (50 mg: Cat. No.: HY-14616. CAS No.: 555-66-8. Molecular Formula: C17H24O3. Molecular Weight: 276.37) were purchased from MedChemTronica European branch of MCE (MedChemExpress) MedChemTronica Sollentuna, Sweden. We also purchased Tween (80: Cat. No.: HY-Y1891. CAS No.: 9005-65-6), dimethyl sulfoxide (DMSO: Cat. No.: HY-Y0320. CAS No.: 67-68-5 Molecular Formula: C2H6OS. Molecular Weight: 78.13), and Phosphate-Buffered Saline (PBS Buffer (1×): Cat. No.: HY-K3005) from the same company. Growth medium: Mueller–Hinton agar MHA and Sabouraud Dextrose Agar (SDA). Microorganism strains: *Staphylococcus aureus* ATCC25923, *Escherichia coli* ATCC25922, *Pseudomonas aeruginosa* ATCC27853, and *Candida albicans* ATCC10231. The step-by-step preparation of disinfectants is presented in the Supplementary File.

### 2.2. Preparation of Chemical Disinfectants

#### 2.2.1. Preparation of Dimethyl Sulfoxide (DMSO) Solution

To prepare a 0.5% DMSO (dimethyl sulfoxide) solution, used to dissolve Zingerone, [6]-Gingerol, and Shogaol, 50 μL of pure DMSO was transferred using a sterile micropipette into a tube [33].

Then, 9.95 mL of the selected diluent PBS was added to a sterile conical 20 mL tube. The solution was mixed gently but thoroughly using vortex mixer vortexing. The container was labeled with 0.5% DMSO, 10 mL, and the date.

#### 2.2.2. Preparation of Tween Solution

Next, 160 μL of pure Tween 80 was transferred using a sterile pipette into a 20 mL sterile container, and 19.84 mL of sterile PBS was subsequently added into the container. The solution was mixed using vortexing, and the container was labeled with “0.8% Tween-80” and the date.

#### 2.2.3. Preparation of 10% Zingerone, 10% [6]-Gingerol, and 10% Shogaol

Using a sterile pipette, 1 mL and 0.5 mL of 0.5% DMSO were transferred into three 1.5 mL Eppendorf tubes; each separate tube was mixed with 100 mg of Zingerone, 100 mg of [6]-Gingerol, and 50 mg Shogaol using vortexing. The containers were subsequently labeled with 10% Zingerone, 10% [6]-Gingerol, and 10% Shogaol.

#### 2.2.4. Preparation of Bacterial and Fungal Suspension

To prepare the bacterial suspension, colonies were transferred using a sterile loop into a sterile tube containing 10 mL of PBS. Based on McFarland standards:

0.5 McFarland ≈ 1.5 × 10^8^ CFU/mL

The starting suspension (assumed) was 1.5 × 10^8^ CFU/mL, and the target suspension was 5.7 × 10^5^ CFU/mL [33].

According to the formula

C1 × V1 = C2 × V,(1)

A total of 4981 μL PBS is mixed with 19 μL of stock concentration, leading to a target suspension concentration of 5.7 × 10^5^.

To prepare the fungal suspension, colonies were transferred using a sterile loop into a sterile tube containing 10 mL of PBS. Based on McFarland standards:

1 McFarland ≈ 1 × 10^7^ CFU/mL

The starting suspension (assumed) was 1 × 10^7^ CFU/mL, and the target suspension was 1 × 10^6^ CFU/mL [30,31].

Using the same formula used for bacteria (i.e., the one mentioned above), 9 mL PBS is mixed with 1 mL of stock concentration, leading to a target suspension concentration of 1 × 10^6^.

### 2.3. Time–Kill Assay with Neutralization

**Figure 1:**
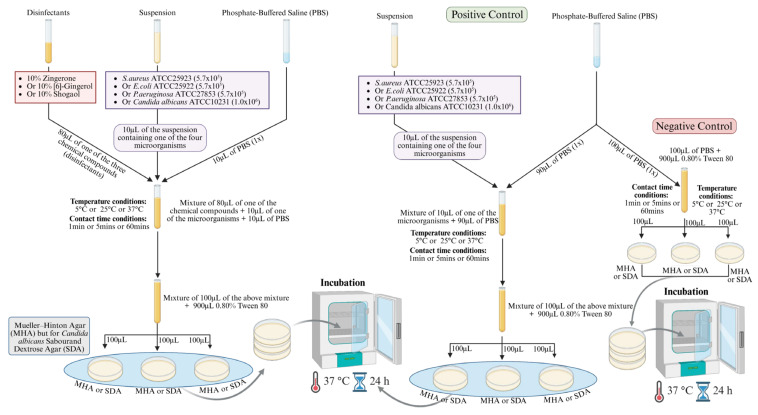
Schematic overview of the time–kill assay procedure to evaluate the antimicrobial effect of chemical disinfectants on bacteria and fungi.

The time–kill assay was performed following the standard methodology [39] with modifications. Figure 1 summarizes the 5 steps involved in testing the efficacy of the disinfectants (10% Zingerone, 10% [6]-Gingerol, and 10% Shogaol) against selected microbes under various temperatures and contact times. The protocol for the reaction mixture involved transferring 80 μL of 10% Zingerone or 10% [6]-Gingerol or 10% Shogaol into separate sterile tubes, followed by the addition of 10 μL of *S. aureus* ATCC25923 or *E. coli* ATCC25922 or *P. aeruginosa* ATCC27853 or *C. albicans* ATCC10231, and 10 μL of PBS (1×). The same protocol was repeated for 3 temperatures and 3 contact times. The reaction mixture was incubated at three different temperatures—5 °C, 25 °C, and 37 °C—and at three different contact times—1 min, 5 min, and 60 min. After the specified contact time at varied temperatures, 100 μL was transferred into a new tube containing 900 μL of 0.8% Tween 80, serving as a neutralizer. Subsequently, 100 μL (from the 1000 μL) is plated on Mueller–Hinton agar (MHA) for bacterial growth or Sabouraud Dextrose Agar (SDA) for fungal growth and incubated at 37 °C for 24 h. The viable count of each microorganism cell after exposure to Zingerone, [6]-Gingerol, and Shogaol was determined by colony-forming units (CFU/mL). The results were calculated by comparing the CFU/mL values of each microorganism before and after exposure to the tested compounds, and the reduction was calculated accordingly.

#### Controls

- Positive control (without disinfectant)

First, 10 μL of the microbial suspension is mixed with 90 μL of PBS in a sterile tube. The reaction is mixed using vortexing and incubated at one of the three different temperatures, 5 °C, 25 °C, or 37 °C, and at one of the three different contact times, 1 min, 5 min, or 60 min. Subsequently, 100 μL of the mixture is transferred into a sterile tube containing 900 μL of 0.8% Tween 80. Finally, 100 μL (from the 1000 μL) is plated on MHA for bacterial growth or SDA for fungal growth and incubated at 37 °C for 24 h.

- Negative control (without microbes)

First, 80 μL of 10% Zingerone, 10% [6]-Gingerol, or 10% Shogaol is transferred into a separate sterile tube and mixed with 20 μL of PBS. The reaction is mixed using vortexing and incubated at one of the three different temperatures, 5 °C, 25 °C, or 37 °C, and at one of the three different contact times, 1 min, 5 min, or 60 min. Subsequently, 100 μL is transferred into a sterile tube containing 900 μL of 0.8% Tween 80. Finally, 100 μL (from the 1000 μL) is plated on MHA for bacterial growth or SDA for fungal growth and incubated at 37 °C for 24 h.

### 2.4. Statistical Analysis

A one-way analysis of variance (ANOVA) was conducted separately for each temperature to determine whether there were statistically significant differences in the mean D-values among the four tested microorganisms. Prior to ANOVA, the assumptions of normality and homogeneity of variances were evaluated using the Shapiro–Wilk test and Levene’s test, respectively. Data were considered normally distributed and variances homogeneous if *p* > 0.05. While the ANOVA results indicated significant differences (*p* < 0.05), post hoc pairwise comparisons were performed using Tukey’s Honestly Significant Difference (HSD) test to identify specific group differences. The results were expressed as mean ± standard deviation (SD), and a significance level of 0.05 was used throughout.

## 3. Results

### 3.1. Evaluation of Antibacterial and Antifungal Activity of 10% Zingerone

The results of the antimicrobial efficacy of 10% Zingerone were evaluated against four representative microorganisms, including *E. coli*, *P. aeruginosa*, *S. aureus*, and *C. albicans*, at three different temperatures (5 °C, 25 °C, and 37 °C) and across various contact times (0, 1, 5, and 60 min) are summarized in Table 1. Overall, the results demonstrated that 10% Zingerone is capable of achieving complete microbial inactivation within 60 min under different temperature conditions. The rate of microbial kill was enhanced at higher temperatures and varied slightly between organisms, with Gram-negative bacteria (*E. coli*) showing slightly faster reductions than *S. aureus*, while *C. albicans* exhibited consistent susceptibility.

#### 3.1.1. Percent Kill After Exposure to 10% Zingerone

**Figure 2:**
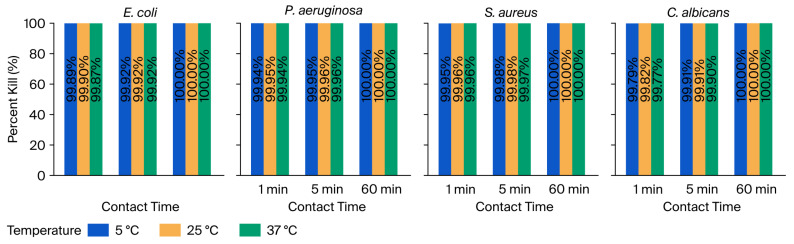
Percent kill of *E. coli*, *P. aeruginosa*, *S. aureus*, and *C. albicans* after exposure to 10% Zingerone at 5 °C, 25 °C, and 37 °C and different contact times.

The antimicrobial efficacy of 10% Zingerone was also evaluated by determining the percent kill of *E. coli*, *P. aeruginosa*, *S. aureus*, and *C. albicans* following exposure at various temperatures and contact times. All organisms exhibited a rapid reduction in viability within the first minute of exposure, with further enhancement over time, culminating in complete inactivation (100%) by 60 min across all temperature conditions, as shown in Figure 2. Overall, the results demonstrated that 10% Zingerone exerts a broad-spectrum and time-dependent antimicrobial effect. Figure 2 clearly illustrates this pattern, with temperature-coded bars representing kill percentages for each organism over the contact time course. The vertical display of exact percent values within each bar further emphasizes the consistent and complete reduction achieved after 60 min.

#### 3.1.2. Decimal Reduction Times (D-Values) After Exposure to 10% Zingerone

The D-values indicate the time required to achieve a 1-log10 (90%) reduction in viable cell counts. The value represents the time required to achieve a 90% reduction in viable microbial count. Lower D-values indicate faster antimicrobial action. The D-values of *E. coli*, *P. aeruginosa*, *S. aureus*, and *C. albicans* following exposure to 10% Zingerone were evaluated across the three temperatures. The results revealed that *P. aeruginosa* exhibited the lowest D-values across all temperatures, ranging from 0.58 min at 37 °C to 0.65 min at 5 °C. *E. coli* followed closely, with D-values ranging from 0.70 to 0.80 min. These findings indicate that Gram-negative bacteria are more susceptible to rapid inactivation by Zingerone under the tested conditions. In contrast, *S. aureus* and *C. albicans* demonstrated significantly higher D-values, ranging from 1.00 to 1.35 min. The longest D-value was observed in *C. albicans* at 5 °C (1.35 min), suggesting greater thermal stability or resistance to the compound at lower temperatures. *S. aureus* showed intermediate D-values, between 1.00 and 1.20 min, across all conditions.

**Figure 3:**
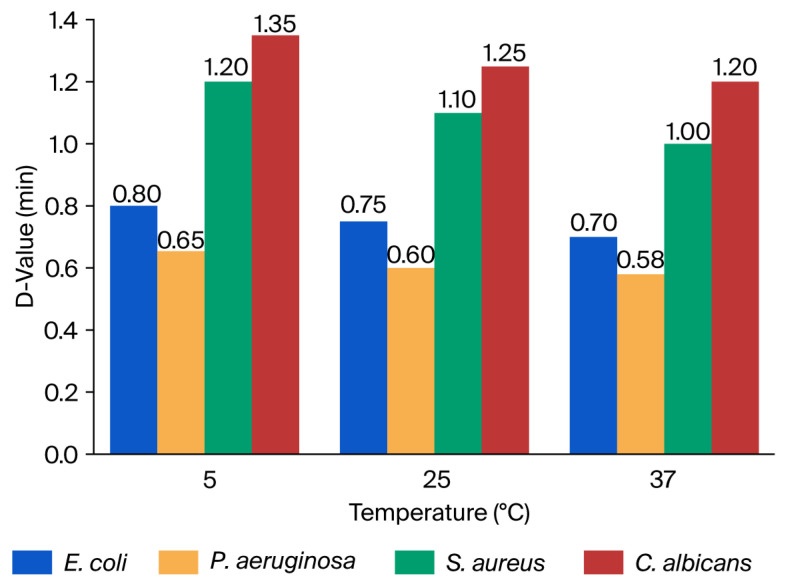
Decimal reduction times (D-values, minutes) of *E. coli*, *P. aeruginosa*, *S. aureus*, and *C. albicans* following exposure to 10% Zingerone at 5 °C, 25 °C, and 37 °C.

A slight temperature-dependent reduction in D-values was observed for all tested organisms, particularly among Gram-negative species. For example, the D-value for *E. coli* decreased from 0.80 min at 5 °C to 0.70 min at 37 °C. This trend suggests enhanced antimicrobial activity of Zingerone at elevated temperatures, possibly due to an increased diffusion rate, better solubility, or greater membrane penetration. The D-values of the tested microbes using Zingerone are presented in Figure 3. Log10 reduction in microorganisms after exposure to 10% Zingerone at different temperatures and contact times is presented in Figure S1 of the Supplementary File. A comparison of D-values, minutes, and 1 min kill percentages of Zingerone against the tested microbes is presented in Figure S2 of the Supplementary File.

### 3.2. Evaluation of Antibacterial and Antifungal Activity of 10% [6]-Gingerol

The antimicrobial activity of 10% [6]-Gingerol was assessed against four microbial strains. Each organism was exposed to 10% [6]-Gingerol at three different temperatures and sampled at four contact times. The results, which are summarized in Table 2, indicate that 10% [6]-Gingerol exerts a rapid and strong antimicrobial effect across a broad range of microbial types. The effect was slightly enhanced at higher temperatures, supporting the temperature-dependent enhancement of its action. These results highlight [6]-Gingerol’s potential as a broad-spectrum antimicrobial agent effective in both bacterial and fungal applications.

#### 3.2.1. Percent Kill After Exposure to 10% [6]-Gingerol

**Figure 4:**
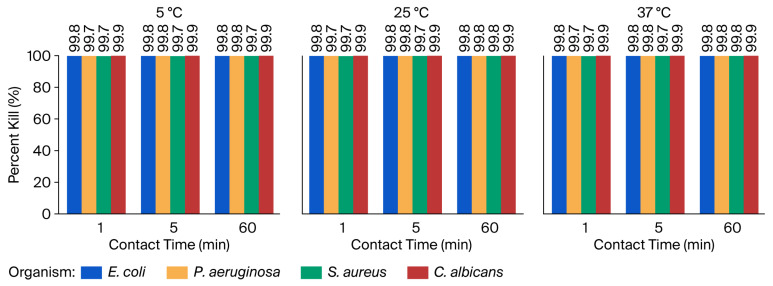
Percent kill of *E. coli*, *P. aeruginosa*, *S. aureus*, and *C. albicans* after exposure to 10% [6]-Gingerol at 5 °C, 25 °C, and 37 °C and different contact times.

As shown in Figure 4, the antimicrobial activity of 10% [6]-Gingerol was evaluated against four test microbes at three different incubation temperatures and contact times. Overall, the figure shows a clustered bar chart illustrating the percent kill of each microorganism at different contact times across the three tested temperatures. *C. albicans* consistently exhibited the highest susceptibility to [6]-Gingerol, especially at 25 °C and 37 °C, suggesting a greater sensitivity of fungal and bacterial strains.

#### 3.2.2. Decimal Reduction Times (D-Values) After Exposure to 10% [6]-Gingerol

**Figure 5:**
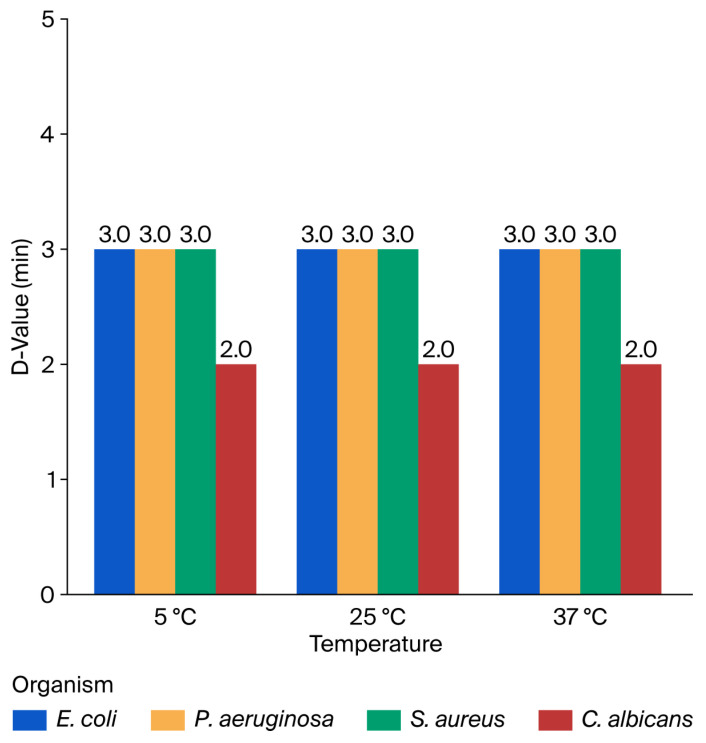
D-values (minutes) of *E. coli*, *P. aeruginosa*, *S. aureus*, and *C. albicans* following exposure to 10% [6]-Gingerol for 0–5 min at 5 °C, 25 °C, and 37 °C.

The decimal reduction time (D-value) was determined for the test microbes following treatment with 10% [6]-Gingerol at three different temperatures (5 °C, 25 °C, and 37 °C), as shown in Figure 5. The D-values for *E. coli*, *P. aeruginosa*, and *S. aureus* were consistent across all three tested temperatures, with values of 3.0 min. This uniformity indicates that temperature had a minimal influence on the early killing kinetics of [6]-Gingerol against these bacterial strains. In contrast, *C. albicans* showed a consistently lower D-value of 2.0 min, demonstrating a more rapid decline in viable fungal cells during the same exposure period. This suggests a higher susceptibility of *C. albicans* to [6]-Gingerol compared to the bacterial strains tested. Log10 reduction in microorganisms after exposure to 10% [6]-Gingerol at different temperatures and contact times is presented in Figure S3 of the Supplementary File. A comparison of D-values, minutes, and 1 min kill percentages of [6]-Gingerol against the tested microbes is presented in Figure S4 of the Supplementary File.

### 3.3. Evaluation of Antibacterial and Antifungal Activity of 10% Shogaol

Table 3 summarizes the antimicrobial activity of 10% Shogaol against four representative microorganisms across three incubation temperatures and over four contact times. Overall, the results indicate that 10% Shogaol demonstrated significant antimicrobial effects, especially against *E. coli* and *C. albicans*, with slightly less pronounced activity against *P. aeruginosa* and *S. aureus*. These findings support the potential application of Shogaol as an antimicrobial agent effective across multiple pathogenic species and a wide temperature range.

#### 3.3.1. Percent Kill After Exposure to 10% Shogaol

**Figure 6:**
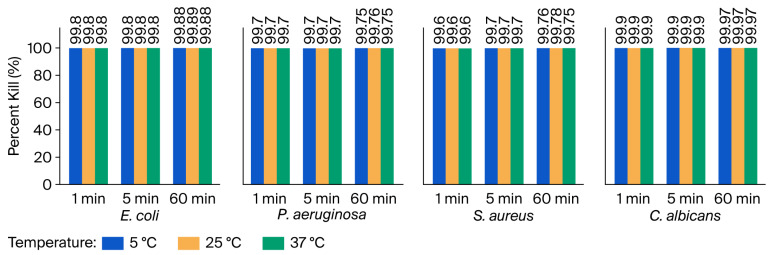
Percent kill of microorganisms following exposure to 10% Shogaol at 5 °C, 25 °C, and 37 °C over contact times of 1, 5, and 60 min.

Figure 6 presents the percent kill of *E. coli*, *P. aeruginosa*, *S. aureus*, and *C. albicans* after treatment with 10% Shogaol. The results demonstrate rapid microbial reductions within the first minute, with enhanced killing at longer contact times and consistent antimicrobial activity irrespective of temperature. The results revealed that *E. coli* and *C. albicans* are highly susceptible to 10% Shogaol, with percent kills exceeding 99.7% within the first minute of exposure across all temperatures. This rapid and potent antimicrobial effect was sustained for 60 min, where the percent kill approached or exceeded 99.9%. *P. aeruginosa* and *S. aureus* showed slightly lower susceptibility, with percent kills ranging from approximately 99.5% to 99.8% after 1 min of exposure, suggesting a moderate level of resistance compared to *E. coli* and *C. albicans*. However, longer contact times consistently improved microbial kill, culminating in reductions exceeding 99.7% at 60 min. Importantly, the antimicrobial efficacy of 10% Shogaol was consistent across the range of temperatures studied, indicating its potential applicability in diverse environmental or clinical settings.

#### 3.3.2. Decimal Reduction Times (D-Values) After Exposure to 10% Shogaol

**Figure 7:**
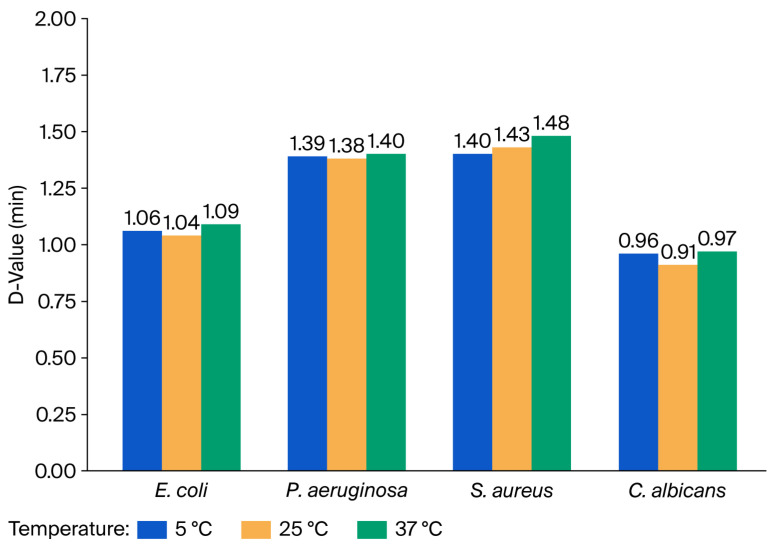
Decimal reduction times (D-values) and one-minute kill percentages of *E. coli*, *P. aeruginosa*, *S. aureus*, and *C. albicans* following exposure to 10% Shogaol at 5 °C, 25 °C, and 37 °C.

To assess the antimicrobial efficacy of 10% Shogaol, D-values were calculated for four clinically significant microorganisms. The results demonstrated that D-values varied across both microbial species and temperatures. Overall, the results suggest that 10% Shogaol is effective across a range of microorganisms and temperatures, with the most pronounced effects observed against *C. albicans* and *E. coli*. The antimicrobial efficacy appeared optimal at 25 °C, particularly for fungal pathogens, though the differences across temperatures were generally subtle. The comparative resistance of *P. aeruginosa* highlights the need for more potent strategies or a combination of disinfectants when targeting this organism. Figure 7 summarizes the D-values of each microorganism at all three temperatures. Log10 reduction in microorganisms after exposure to 10% Shogaol at different temperatures and contact times is presented in Figure S5 of the Supplementary File. A comparison of D-values, minutes, and 1 min kill percentages of Shogaol against the test microbes is presented in Figure S6 of the Supplementary File.

### 3.4. Statistical Analysis

ANOVA was performed to compare the D-values and 1 min kill percentages of four representative microorganisms after exposure to 10% Zingerone, [6]-Gingerol, and Shogaol across various temperatures. The results indicated that, after exposure to 10% Zingerone, the D-values varied significantly among the microorganisms at each temperature, with *P. aeruginosa* generally showing the lowest D-values (highest susceptibility), and *S. aureus* and *C. albicans* demonstrating higher D-values. The 1 min kill percentages were consistently high (>99.7%) across all organisms and temperatures, reflecting rapid microbial inactivation.

After exposure to 10% [6]-Gingerol, the results indicated a highly significant difference between groups (F(3, 8) = 1.01 × 1031, *p* < 0.0001), indicating that the microbial response to [6]-Gingerol varied significantly by species. Tukey’s HSD test confirmed that *C. albicans* exhibited significantly lower D-values than all bacterial strains (*p* < 0.001), highlighting its greater sensitivity to 10% [6]-Gingerol. In contrast, no significant differences were found among *E. coli*, *P. aeruginosa*, and *S. aureus*, indicating a consistent bactericidal effect of the compound across these organisms.

The results revealed a statistically significant difference among the tested organisms (F(3,8) = 118.83, *p* < 0.0001) after exposure to 10% Shogaol, indicating that microbial susceptibility to Shogaol varied significantly based on species. To further examine these differences, Tukey’s Honestly Significant Difference (HSD) post hoc test was applied. The test confirmed that *C. albicans* exhibited significantly higher 1 min kill percentages than all bacterial species (*p* < 0.01), reflecting its high sensitivity to Shogaol. In contrast, *P. aeruginosa* demonstrated the lowest kill rates, with statistically significant differences from *C. albicans*, *E. coli*, and *S. aureus* (*p* < 0.05). Although *E. coli* and *S. aureus* showed similar mean kill percentages, their values were still statistically distinct from *C. albicans* and *P. aeruginosa*.

## 4. Discussion

The chemical structure of ginger-derived compounds plays a vital role in their antimicrobial potency. The key differences between these compounds lie in the functional groups on their side chain, which directly influence their reactivity, hydrophobicity, and ability to interact with microbial cellular components. For example, Zingerone, a hydrogenated metabolite, has a saturated side chain, while Gingerol, the primary pungent compound in fresh ginger, possesses a β-hydroxy keto group, and Shogaol (which forms as a result of the dehydration of Gingerol) possesses an α, β-unsaturated carbonyl moiety. Thus, these basic structural variations determined their distinct mechanisms and efficacy [40,41,42].

The mode of action of these compounds also varies due to differences in their chemical structure. The mechanism of action of Zingerone relies on its ability to integrate into membranes due to its hydrophobicity, causing fluidity changes and mild disruption [43]. The mechanism of action of Gingerol relies more on its phenolic hydroxyl group, which can act as a pro-oxidant, thereby generating reactive oxygen species that cause oxidative stress in microbial cells. Unlike Shogaol, Gingerol lacks a strong Michael acceptor system; however, it can also disrupt membrane integrity, albeit less effectively compared with Shogaol due to its higher polarity from the β-hydroxy group [42]. The α, β-unsaturated carbonyl group in Shogaol acts as a Michael acceptor, making it a highly reactive electrophile. This chemical structure readily forms covalent bonds with nucleophilic thiol (-SH) groups in cysteine residues of microbial proteins and enzymes. This non-specific, irreversible binding leads to simultaneous disruption of multiple essential cellular functions (i.e., energy metabolism, cell wall biosynthesis, and enzyme activity) [44].

The evaluation of the antimicrobial efficacy of Zingerone has shown that it is effective against all the test microbes, with percent kill values of over 99% at 5 °C, 25 °C, and 37 °C, after 1 min and 5 min, and 100% after 60 min (as shown in Table 1 and Figure 2).

A comparative analysis between the test microbes indicated that Zingerone is more effective against Gram-negative bacteria (*E. coli* and *P. aeruginosa*) compared with *S. aureus* (Gram-positive bacterium). On the contrary, [26] reported the susceptibility of natural ginger extract (standardized to gingerol content) against Gram-positive bacteria compared with Gram-negative bacteria. The study opted for disk diffusion, MIC, and biofilm inhibition to evaluate the antimicrobial activity of natural ginger extracts against *S. aureus*, *Bacillus cereus*, *E. coli*, *P. aeruginosa*, *Salmonella Typhi*, and *C. albicans*. The results also indicated that Zingerone is more effective against bacteria than fungi.

Nonetheless, the assessment of the antimicrobial efficacy of [6]-Gingerol has shown that it exerts a rapid and strong antimicrobial effect across all the tested microbes, with percent kill values of over 99.6% after 1 min. However, unlike in Zingerone, the total kill was not recorded after prolonged exposure (60 min), with a percent kill of over 99.7% (as shown in Table 2 and Figure 4). The effect of 6-Gingerol on *E. coli*, *P. aeruginosa*, *S. aureus*, and *C. albicans* at 250 C after 1, 5, and 60 min is presented in Figure S7 of the Supplementary File. The comparative analysis between the test microbes indicated that [6]-Gingerol is more effective against *C. albicans* (fungi) than bacteria. Moreover, a comparison between Gram-positive and Gram-negative bacteria has shown that [6]-Gingerol is more effective against Gram-negative bacteria (*E. coli* and *P. aeruginosa*) compared with *S. aureus* (Gram-positive bacterium). On the contrary, the study reported by [20] indicated that *S. aureus* (Gram-positive) is more susceptible than Gram-negative bacteria, with the highest inhibition at 30 mg/mL (20 mm). The study assesses antimicrobial activity (based on disk diffusion–inhibition zone) of naturally derived gingerol (0.1–0.3% *w*/*v*, 37 °C, and after 24 h) against vaginitis-associated microbes (*S. aureus*, *E. coli*, *Klebsiella* spp., *C. albicans*, *C. tropicalis*, *C. krusei*) in comparison with antibiotics. Similarly, the study reported by [27] revealed that commercially sourced [6]-Gingerol synergies with antibiotics showed stronger activity against Gram-positive bacteria than Gram-negative bacteria. The study tested the efficacy of [6]-Gingerol (37 °C, time–kill up to 24 h) against *S. aureus* (including MRSA), *E. coli*, and *P. aeruginosa*.

Consequently, the evaluation of the antimicrobial efficacy of Shogaol has shown that it is effective against the test microbes, with a percent kill of over 95% within the first minute of exposure. A similar trend with [6]-Gingerol was also recorded for prolonged exposure (as shown in Table 3 and Figure 6). A comparative analysis between the tested microbes indicated that Shogaol is more effective against *E. coli* and *C. albicans*, with percent kills exceeding 99.7% within the first minute of exposure. This rapid and potent antimicrobial effect was sustained for 60 min, where the percent kill approached or exceeded 99.9%. The potent antifungal and antibiofilm activity of 6-Shogaol against *Candida* spp., especially *C. auris*, is also reported by [45]. The study evaluated the antifungal activity of 6-Shogaol (37 °C, time–kill up to 24 h) against *C. auris*, *C. albicans*, and *C. tropicalis* using broth dilution, biofilm inhibition, and time–kill.

Based on the evaluation of the impact of temperature (5 °C, 25 °C, 37 °C) and contact time (1, 5, 60 min) on antimicrobial efficacy, the result has shown that the rate of antimicrobial efficacy of Zingerone was enhanced at higher temperatures and varied slightly between organisms, with *E. coli* (Gram-negative bacteria) exhibiting slightly faster reductions than *S. aureus* (Gram-positive bacteria) and *C. albicans* (fungi), showing consistent susceptibility. In the case of [6]-Gingerol (as shown in Table 2), the antimicrobial efficacy increased with the increase in temperature, while in the case of Shogaol (as shown in Table 3), temperature variations had minimal impact on the antimicrobial activity. Based on contact time, Zingerone is more effective, reaching 100% kill after 60 min compared with [6]-Gingerol and Shogaol.

## 5. Conclusions

The emergence and re-emergence of pathogenic microbes continue to impose a burden on the global healthcare sector, and despite numerous antimicrobial agents, the surge in microbial resistance to synthetic disinfectants continues to pose a threat, necessitating intensified research on chemically synthesized natural bioactive compounds. In this study, we investigate how temperature modulates the antimicrobial efficacy of Zingerone, [6]-Gingerol, and Shogaols against *S aureus*, *E. coli*, *P. aeruginosa*, and *C. albicans*, with findings indicating that 10% Zingerone demonstrated effective and rapid antimicrobial action against both bacterial and fungal species, [6]-Gingerol demonstrated rapid and strong antimicrobial activity with temperature-independent early-phase efficacy, and Shogaol demonstrated strong activity under all temperature conditions with optimal performance at 25 °C. Future studies will attempt to evaluate the efficacy of the three disinfectants on other Gram-positive and Gram-negative bacteria, such as *Salmonella enteritidis*, *Salmonella typhimurium*, *Bacillus cereus*, *Listeria monocytogenes*, and *Enterococcus faecalis*, as well as other fungi, including *Candida auris*, *Aspergillus flavus*, and *Cryptococcus neoformans*.

## 6. Patents

The patent numbers obtained for this work are listed below:

Zingerone Application No. 2025/009718;

[6]-Gingerol Application No. 2025/009721;

Shogaol Application No. 2025/009723.

## Figures and Tables

**Table 1:** Microbial counts (CFU/mL) after exposure to 10% Zingerone at contact times of 0, 1, 5, and 60 min.

Microbes/Mean ± SD	Temperature (°C)	0 min	1 min	5 min	60 min
*E. coli*	5	5.7 × 10^5^	3.4 × 10^2^	2.7 × 10^2^	0
	25	5.7 × 10^5^	3.0 × 10^2^	2.5 × 10^2^	0
	37	5.7 × 10^5^	3.2 × 10^2^	2.4 × 10^2^	0
Mean ± SD		5.70 ± 0.00 × 10^5^	3.20 ± 0.20 × 10^2^	2.53 ± 0.15 × 10^2^	0
*P. aeruginosa*	5	5.7 × 10^5^	2.6 × 10^2^	1.3 × 10^2^	0
	25	5.7 × 10^5^	2.0 × 10^2^	1.0 × 10^2^	0
	37	5.7 × 10^5^	2.4 × 10^2^	1.6 × 10^2^	0
Mean ± SD		5.70 ± 0.00 × 10^5^	2.33 ± 0.31 × 10^2^	1.30 ± 0.30 × 10^2^	0
*S. aureus*	5	5.7 × 10^5^	1.2 × 10^3^	5.3 × 10^2^	0
	25	5.7 × 10^5^	1.0 × 10^3^	5.0 × 10^2^	0
	37	5.7 × 10^5^	1.3 × 10^3^	5.5 × 10^2^	0
Mean ± SD		5.70 ± 0.00 × 10^5^	1.17 ± 0.15 × 10^3^	5.27 ± 0.25 × 10^2^	0
*C. albicans*	5	1 × 10^6^	1.1 × 10^3^	7.8 × 10^2^	0
	25	1 × 10^6^	1.0 × 10^3^	7.5 × 10^2^	0
	37	1 × 10^6^	1.3 × 10^3^	7.7 × 10^2^	0
Mean ± SD		1.00 ± 0.00 × 10^6^	1.13 ± 0.15 × 10^3^	7.67 ± 0.15 × 10^2^	

**Table 2:** Microbial counts (CFU/mL) after exposure to 10% [6]-Gingerol at contact times of 0, 1, 5, and 60 min.

Microbes/Mean ± SD	Temperature (°C)	0 min	1 min	5 min	60 min
*E. coli*	5	5.7 × 10^5^	1.2 × 10^3^	1.1 × 10^3^	9.5 × 10^2^
	25	5.7 × 10^5^	1.1 × 10^3^	1.0 × 10^3^	9.0 × 10^2^
	37	5.7 × 10^5^	1.4 × 10^3^	1.1 × 10^3^	9.4 × 10^2^
Mean ± SD		5.70 ± 0.00 × 10^5^	1.23 ± 0.15 × 10^3^	1.07 ± 0.06 × 10^3^	9.30 ± 0.25 × 10^2^
*P. aeruginosa*	5	5.7 × 10^5^	1.5 × 10^3^	1.3 × 10^3^	9.8 × 10^2^
	25	5.7 × 10^5^	1.4 × 10^3^	1.2 × 10^3^	9.5 × 10^2^
	37	5.7 × 10^5^	1.5 × 10^3^	1.3 × 10^3^	9.7 × 10^2^
Mean ± SD		5.70 ± 0.00 × 10^5^	1.47 ± 0.06 × 10^3^	1.27 ± 0.06 × 10^3^	9.67 ± 0.15 × 10^2^
*S. aureus*	5	5.7 × 10^5^	1.8 × 10^3^	1.7 × 10^3^	1.4 × 10^3^
	25	5.7 × 10^5^	1.6 × 10^3^	1.5 × 10^3^	1.4 × 10^3^
	37	5.7 × 10^5^	1.7 × 10^3^	1.6 × 10^3^	1.4 × 10^3^
Mean ± SD		5.70 ± 0.00 × 10^5^	1.70 ± 0.10 × 10^3^	1.60 ± 0.10 × 10^3^	1.40 ± 0.00 × 10^3^
*C. albicans*	5	1 × 10^6^	1.2 × 10^3^	9.8 × 10^2^	5.9 × 10^2^
	25	1 × 10^6^	1.0 × 10^3^	9.0 × 10^2^	5.7 × 10^2^
	37	1 × 10^6^	1.2 × 10^3^	9.6 × 10^2^	6.0 × 10^2^
Mean ± SD		1.00 ± 0.00 × 10^6^	1.13 ± 0.12 × 10^3^	9.47 ± 0.42 × 10^2^	5.87 ± 0.15 × 10^2^

**Table 3:** Microbial counts (CFU/mL) after exposure to 10% Shogaol at contact times of 0, 1, 5, and 60 min.

Microbes/Mean ± SD	Temperature (°C)	0 min	1 min	5 min	60 min
*E. coli*	5	5.7 × 10^5^	1.28 × 10^3^	9.5 × 10^2^	6.8 × 10^2^
	25	5.7 × 10^5^	1.22 × 10^3^	9.2 × 10^2^	6.3 × 10^2^
	37	5.7 × 10^5^	1.30 × 10^3^	9.8 × 10^2^	7 × 10^2^
Mean ± SD		5.70 ± 0.00 × 10^5^	1.27 ± 0.04 × 10^3^	9.50 ± 0.30 × 10^2^	6.70 ± 0.36 × 10^2^
*P. aeruginosa*	5	5.7 × 10^5^	1.90 × 10^3^	1.76 × 10^3^	1.40 × 10^3^
	25	5.7 × 10^5^	1.86 × 10^3^	1.71 × 10^3^	1.36 × 10^3^
	37	5.7 × 10^5^	1.95 × 10^3^	1.80 × 10^3^	1.43 × 10^3^
Mean ± SD		5.70 ± 0.00 × 10^5^	1.90 ± 0.05 × 10^3^	1.76 ± 0.05 × 10^3^	1.40 ± 0.04 × 10^3^
*S. aureus*	5	5.7 × 10^5^	2.1 × 10^3^	1.95 × 10^3^	1.34 × 10^3^
	25	5.7 × 10^5^	2 × 10^3^	1.92 × 10^3^	1.25 × 10^3^
	37	5.7 × 10^5^	2.3 × 10^3^	1.97 × 10^3^	1.40 × 10^3^
Mean ± SD		5.70 ± 0.00 × 10^5^	2.13 ± 0.15 × 10^3^	1.95 ± 0.03 × 10^3^	1.33 ± 0.08 × 10^3^
*C. albicans*	5	1 × 10^6^	1.2 × 10^3^	9.0 × 10^2^	3.1 × 10^2^
	25	1 × 10^6^	1.1 × 10^3^	8.8 × 10^2^	3.0 × 10^2^
	37	1 × 10^6^	1.5 × 10^3^	9.5 × 10^2^	3.4 × 10^2^
Mean ± SD		1.00 ± 0.00 × 10^6^	1.27 ± 0.21 × 10^3^	9.10 ± 0.36 × 10^2^	3.17 ± 0.21 × 10^2^

## Data Availability

The original contributions presented in this study are included in the article.

## Supplementary Materials

The following supporting information can be downloaded at: https://www.mdpi.com/article/10.3390/microorganisms14030539/s1. Figure S1: Log10 Reduction in microorganisms after exposure to 10% Zingerone at different temperatures and contact times. Figure S2: Comparison of decimal reduction times (D-values, minutes) and 1 min kill percentages of *E. coli*, *P. aeruginosa*, *S. aureus*, and *C. albicans* following exposure to 10% Zingerone at 5 °C, 25 °C, and 37 °C. Figure S3. Log10 reduction in microorganisms after exposure to 10% [6]-Gingerol at different temperatures and contact times. Figure S4: Comparison of decimal reduction times (D-values, minutes) and 1 min kill percentages of *E. coli*, *P. aeruginosa*, *S. aureus*, and *C. albicans* following exposure to 10% [6]-Gingerol at 5 °C, 25 °C, and 37 °C. Figure S5. Log10 reduction in microorganisms after exposure to 10% Shogaol at different temperatures and contact times. Figure S6: Comparison of decimal reduction times (D-values, minutes) and 1 min kill percentages of *E. coli*, *P. aeruginosa*, *S. aureus*, and *C. albicans* following exposure to 10% Shogaol at 5 °C, 25 °C, and 37 °C. Figure S7. The effect of 6-Gingerol on *E. coli*, *P. aeruginosa*, *S. aureus*, and *C. albicans* at 25 °C after 1, 5, and 60 min.

## Abbreviations

The following abbreviations are used in this manuscript:

AMR	Antimicrobial Resistance
ANOVA	Analysis of Variance
CFUs	Colony-Forming Units
DMSO	Dimethyl Sulfoxide
HSD	Honestly Significant Difference
MHA	Mueller–Hinton Agar
PBS	Phosphate-Buffered Saline
QACs	Quaternary Ammonium Compounds
SDA	Sabouraud Dextrose Agar
SGDDs	Synthetic Ginger-Derived Disinfectants
