# Supplementary material for: The Effect of Variations in Temperature and Contact Time of Zingerone, [6]-Gingerol and Shogaol as Disinfectants on Staphylococcus aureus, Escherichia coli, Pseudomonas aeruginosa and Candida albicans
Source: Microorganisms. 2026 Feb 26;14(3):539. doi: 10.3390/microorganisms14030539 (PMC13028615; doi:10.3390/microorganisms14030539)
Supplement: Supplementary file 1 [file microorganisms-14-00539-s001.zip › microorganisms-4123066-supplementary.pdf]

Supplementary File

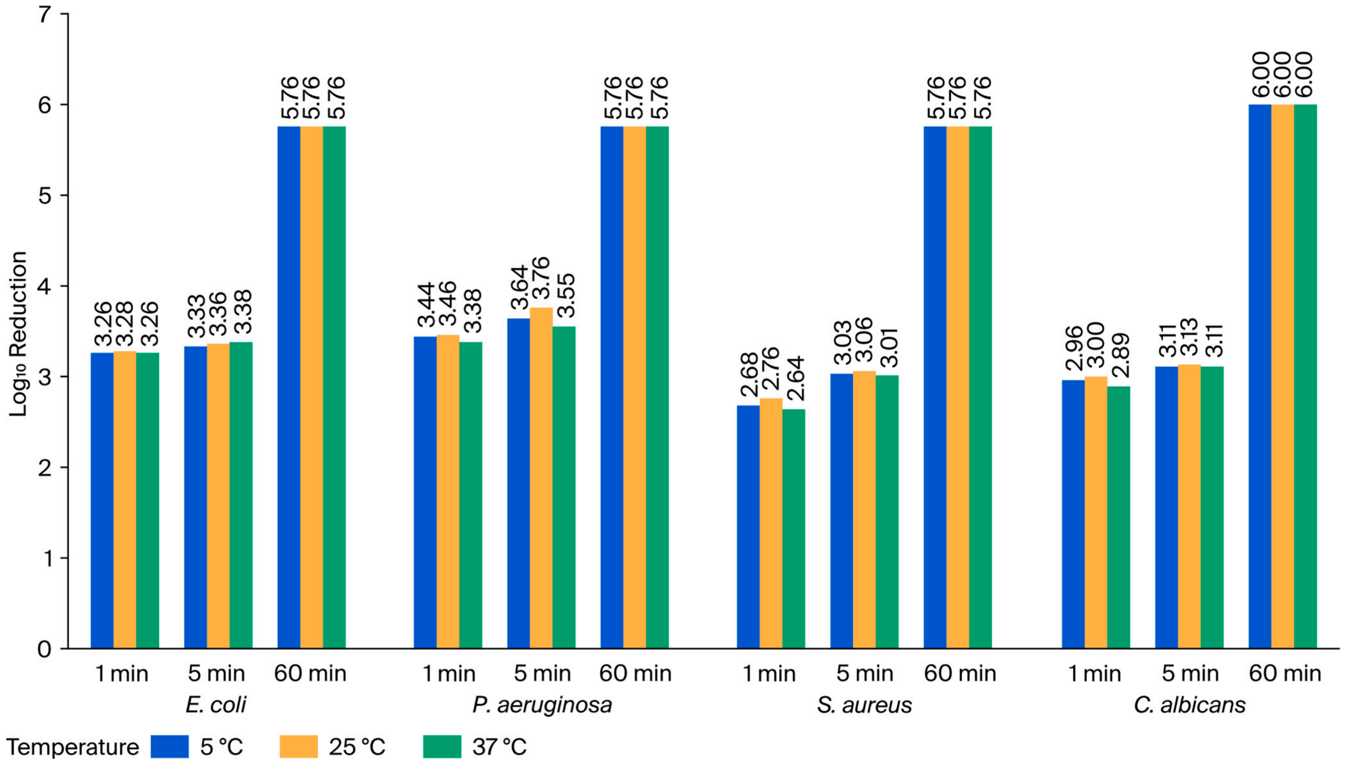

Figure S1. Log10 reduction in viable cell counts of four microorganisms, E. *coli*, *P. aeruginosa*, *S. aureus* and *C. albicans*, after exposure to 10% Zingerone at 5 °C, 25 °C and 37 °C over contact times of 1, 5 and 60 minutes.

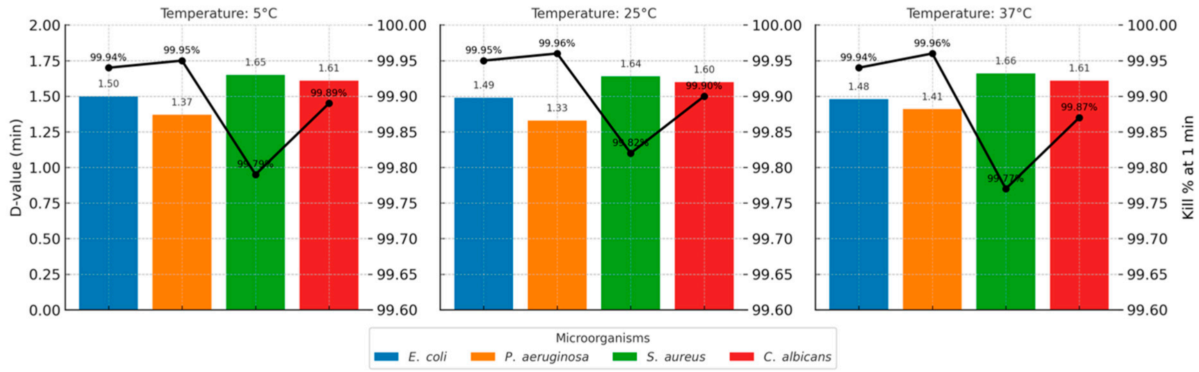

Figure S2. Comparison of decimal reduction times (D-values, minutes) and 1-minute kill percentages of E. coli, P. aeruginosa, S. aureus, and C. albicans following exposure to 10% Zingerone at 5 °C, 25 °C, and 37 °C.

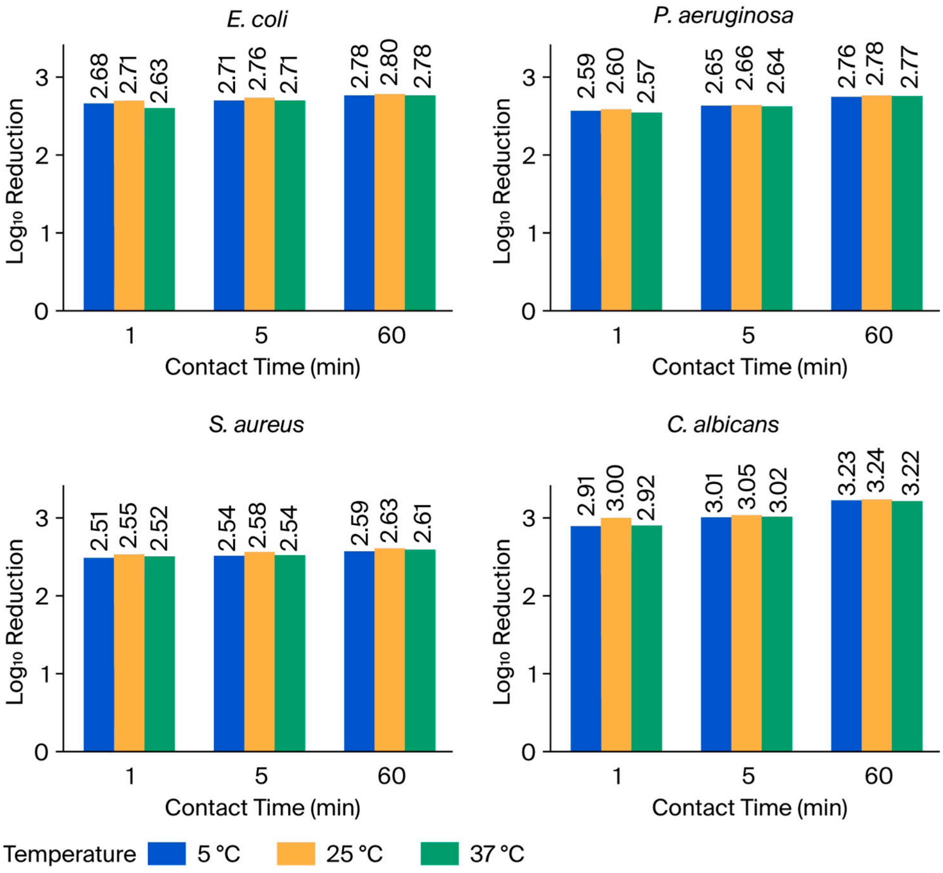

Figure S3. Log10 reduction in microorganisms after exposure to 10% [6]-Gingerol at different temperatures and contact times.

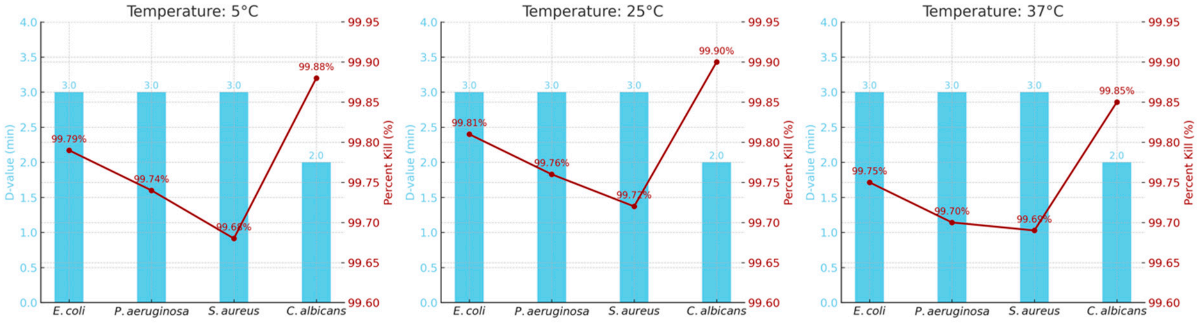

Figure S4. Comparison of decimal reduction times (D-values, minutes) and 1-minute kill percentages of E. coli, P. aeruginosa, S. aureus, and C. albicans following exposure to 10% 6-Gingerol at 5 °C, 25 °C, and 37 °C.

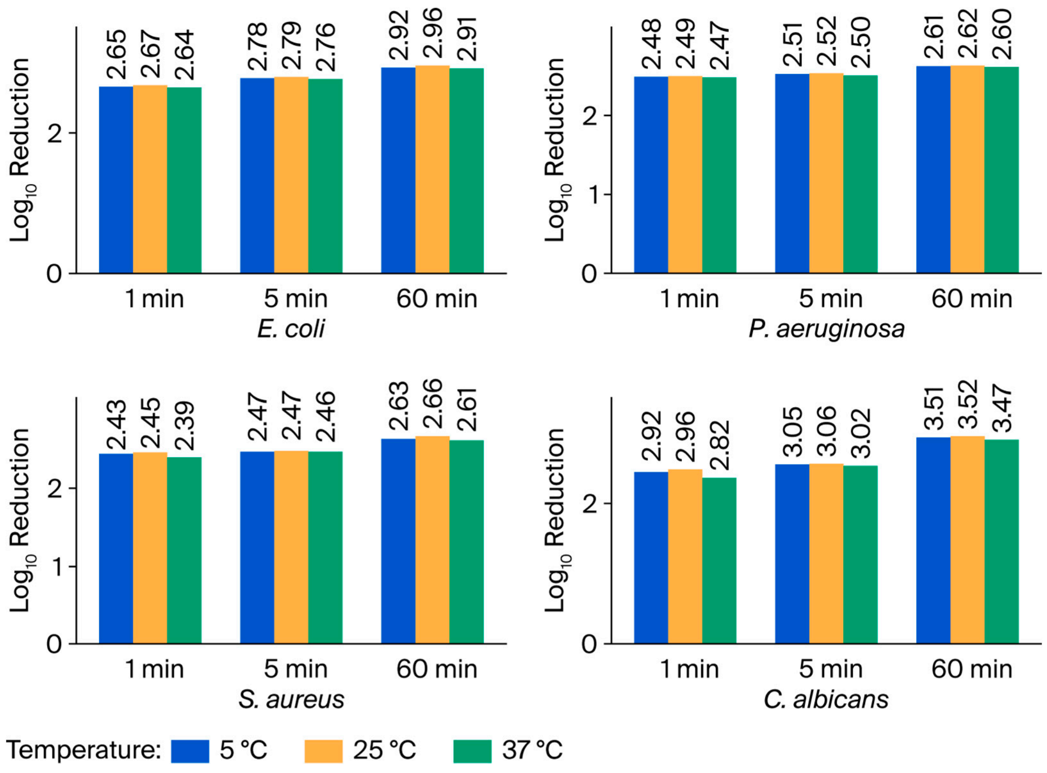

Figure S5. Log10 reduction in microorganisms after exposure to 10% Shogaol at different temperatures and contact times.

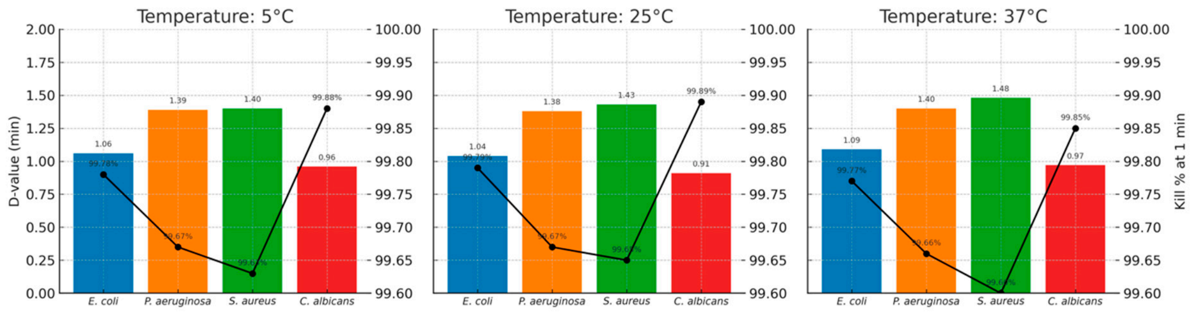

Figure S6. Comparison of decimal reduction times (D-values, minutes) and 1-minute kill percentages of E. coli, P. aeruginosa, S. aureus, and C. albicans following exposure to 10% Shogaol at 5 °C, 25 °C, and 37 °C.

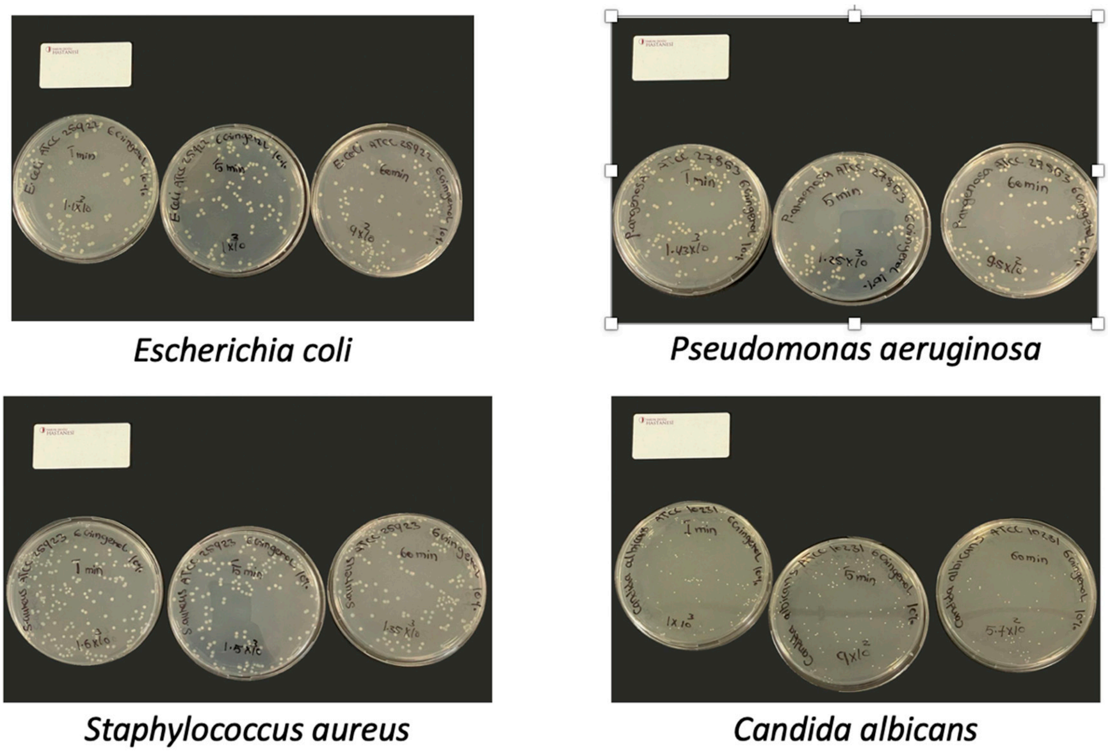

Figure S7. The Effect of 6-Gingerol on *E. coli, P. aeruginosa, S. aureus* and *C. albican* at 25^0 C after 1,5 and 60 minutes
